# Exploring the impacts of social media and crowdsourcing on disaster resilience

**DOI:** 10.12688/openreseurope.13721.1

**Published:** 2021-06-01

**Authors:** Nathan Clark, Kees Boersma, Sara Bonati, Chiara Fonio, Simon Gehlhar, Therese Habig, Richard Lüke, Stefano Morelli, Anne Bach Nielsen, Antonio Opromolla, Veronica Pazzi, Emmanuel Raju

**Affiliations:** 1 VRIJE UNIVERSITEIT AMSTERDAM, Amsterdam, The Netherlands; 2 UNIVERSITÀ DEGLI STUDI DI FIRENZE, Firenze, Italy; 3 SAFETY INNOVATION CENTER E.V., Paderborn, Germany; 4 Global Health Section, Copenhagen Centre For Disaster Research, University of Copenhagen, Copenaghen, Denmark; 5 Link Campus University, Rome, Italy

**Keywords:** Social media, crowdsourcing, disasters, societal resilience, vulnerability

## Abstract

Social media and crowdsourcing (SMCS) are increasingly proving useful for addressing the effects of natural and human-made hazards. SMCS allow different stakeholders to share crucial information during disaster management processes and to strengthen community resilience through engagement and collaboration. To harvest these opportunities there is a need for better knowledge on SMCS for diverse disaster scenarios. These challenges are being addressed within the LINKS Horizon 2020 project. The project aims at strengthening societal resilience by producing advanced learning on the use of SMCS in disasters. This is done through an in-depth study across three knowledge domains (disaster risk perception and vulnerability, disaster management processes, disaster community technologies), the establishment of an interactive Framework, and an online platform in which a community of relevant stakeholders can learn and share knowledge and experiences. This paper provides an overview of the project objectives and approaches and a summary of the initial results.

## Introduction

During the various phases of disaster and crisis management, formal authorities and responding organizations are increasingly looking for meaningful information, knowledge and input from a wide variety of stakeholders, including the private sector, non-governmental organizations, interest groups, local communities and citizens networks. In recent years, regions, states, and municipalities have increasingly worked to integrate social media and crowdsourcing (SMCS) services and technologies into crisis management, be it based on local activities, or globally connected ( Harrison & Johnson, 2019; Riccardi, 2016). Numerous platforms have been built, implemented and used in various disaster contexts and in various parts of the world in order to facilitate crowdsourcing. Such platforms include Ushahidi, Open Street Maps, Crisis Tracking, Ready2Help, and Digital Humanitarian Networks ( Meier, 2015; Rogstadius *et al*., 2013; Schmidt *et al*., 2018). 

For more than a decade, research has been conducted on the support, implementation and use of SMCS, with a focus on the development and implications of new technologies, procedures and applications for gathering and sharing information within communities, and for collaboratively coping with crises. Crowd sourcing can be seen as “the act of taking a job traditionally performed by a designated agent (usually an employee) and outsourcing it to an undefined, generally large group of people in the form of an open call” ( Howe, 2006). Estellés-Arolas & González-Ladrón-de-Guevara (2012, p. 197) defined crowd sourcing as “a type of participative online activity in which an individual, an institution, a non-profit organization, or company proposes to a group of individuals of varying knowledge, heterogeneity, and number, via a flexible open call, the voluntary undertaking of a task.”. They furthermore recognize the reward of undertaking the tasks for the crowd, e.g. citizens, as it contributes to knowledge and experience, self-esteem and resources, and the benefit for the crowd sourcer in terms of the utilization of what the user brought to the table. Crowd sourcing indeed entails mutual benefit.

Research has been undertaken on crowdsourcing methods and tools ( Poblet *et al.*, 2018). Crowd sourcing has been studied in relation to the (lack of) trust in the information exchanged via social media and crowdsourcing at times of disasters ( Mehta *et al.*, 2017), for disaster awareness ( Rogstadius *et al*., 2013), for early warning systems ( Meissen & Fuchs-Kittowski, 2014), digital volunteers ( Starbird, 2011; Zook *et al*., 2010), and rapid damage assessment ( Yuan & Liu, 2018). Recently, the scope of the research has been widened to understand the role of crowdsourcing in disaster risk reduction ( Kankanamge *et al*., 2019) and disaster resilience ( Song *et al*., 2020). Recent technological developments, including social media applications, online interactive platforms and other smart technologies potentially enable crowdsourcing to result in aggregated information from a huge variety of citizens that can enhance professional knowledge and inform both crisis and disaster management and crisis and risk communication ( Boersma *et al*., 2019). 

However, the *effectiveness* of the uses of SMCS in disasters remains unclear owing to the diversity among disaster risk perception and vulnerability (DRPV), disaster management processes (DMP), and disaster community technologies (DCT). The challenge faced by first responders, public authorities and citizens is the absence of common methods, tools and guidelines for effectively understanding and applying SMCS for improved disaster resilience under diverse conditions. What is required is a standard Framework of best (as well as good and bad) practices and community platforms, for producing sustainable advanced learning on the effective use of SMCS in disasters. This challenge is the basis for the work in the LINKS Horizon 2020 project. 

This paper provides insights into the ongoing research and findings of the LINKS project. The overall objective of LINKS is strengthening the *links* between technologies and society for improved European disaster resilience, by producing sustainable advanced learning on the use of SMCS in disasters. The sections in this paper proceed as follows: “The LINKS project” provides an introduction to LINKS and the objectives of project. “The LINKS approach” provides an overview of the core concepts and approaches to the research. “LINKS preliminary findings” summarizes the project’s findings to date, particularly relating to studies across the three knowledge domains of DRPV, DMP, and DCT. The final section of the paper provides a conclusion and lays out the plans to for the work to be done in the coming phases of the project.

## Protocol

### The LINKS project

The LINKS project began in June 2020, funded by the European Commission under the Horizon 2020 Research and Innovation Programme, and in particular under the call Security - Disaster Resilient Society: “Human factors, and social, societal, and organizational aspects for disaster-resilient societies”. LINKS intends to strengthen societal resilience by contributing to a better understanding of the uses of SMCS in disasters. In LINKS, resilience is both a normative and positive quality of a system, institution or individual that increases the capacity to manage disaster risk. LINKS contributes to this process in the context of sustainable advanced learning, as learning is a fundamental aspect of the strengthening of resilience. LINKS defines sustainable advanced learning as a maintainable and evolving collection of knowledge and best practices produced for and by relevant stakeholders. Importantly, sustainable advanced learning entails a cognitive dimension (the capability to gain in-depth knowledge of crises and crisis management, for example), a social dimension (the collaborative efforts to implement that knowledge into new practices), and a transformative dimension whereby reflections are made on how knowledge was learned, what has changed in the process, and how and in what ways new knowledge might continue to evolve. This idea is embedded in the design of the research and outputs of the project. 

Moreover, the project aims to develop sustainable advanced learning on SMCS in disasters, through three sub-objectives:

- achieving a consolidating understanding of SCMS in disasters;
- governing the diversity, emerging from different knowledge domains, of SMCS in disasters;
- connecting multidisciplinary stakeholders in the SCMS/disaster domain to exchange and produce knowledge.

The approaches to these objectives in LINKS are described in the following sections. What is important here is highlighting the actors whom the project is addressing, who are considered at the same time as the target audience, as stakeholders to involve during many steps of the project itself, and as future end users of the project outcomes. These include:

- practitioners (local, national, and European civil protection agencies, first responders, non-governmental organizations, security networks), who need to know and can provide feedback on how SMCS can be integrated into the technical solutions they already use in their work;
- policy and decision makers (local, national, and European agencies and organizations, public authorities, standardization bodies), who have the responsibility to take decisions on how the disaster management processes can be improved through new solutions;
- research networks (research institutions and scientific communities), who can give validity to the research processes and outputs of LINKS;
- industrial bodies (individual companies and local business networks and suppliers of goods and services), who can be engaged in disaster resilience efforts and provide goods or services that can be used for SMCS, crisis management or another relevant interest for LINKS;
- citizens (civil society organizations, educational institutions, vulnerable groups, social movement organizations), who not only need to be protected in disasters, but can also offer active participation, collaboration and valuable contributions in these situations.

The partners who are working on the LINKS project have a wide range of experience and expertise in the areas of disaster management and governance. They reflect the actors to whom the project is addressed, representing: EU emergency management and security organizations and networks; local and national first responders, civil protection and law enforcement agencies; citizens, public authorities and civil society organizations; business communities and industry; and research institutions. 

### The LINKS approach

In order to reach the objectives defined in the previous section, LINKS partners are working on three main areas:

- area A: Assessment of DRPV, DMP, and DCT, and establishment of the knowledge base for the project;
- area B: Development and evaluation of the LINKS Framework;
- area C: Establishment and management of the LINKS Community and LINKS Community Center.

**Figure 1.**
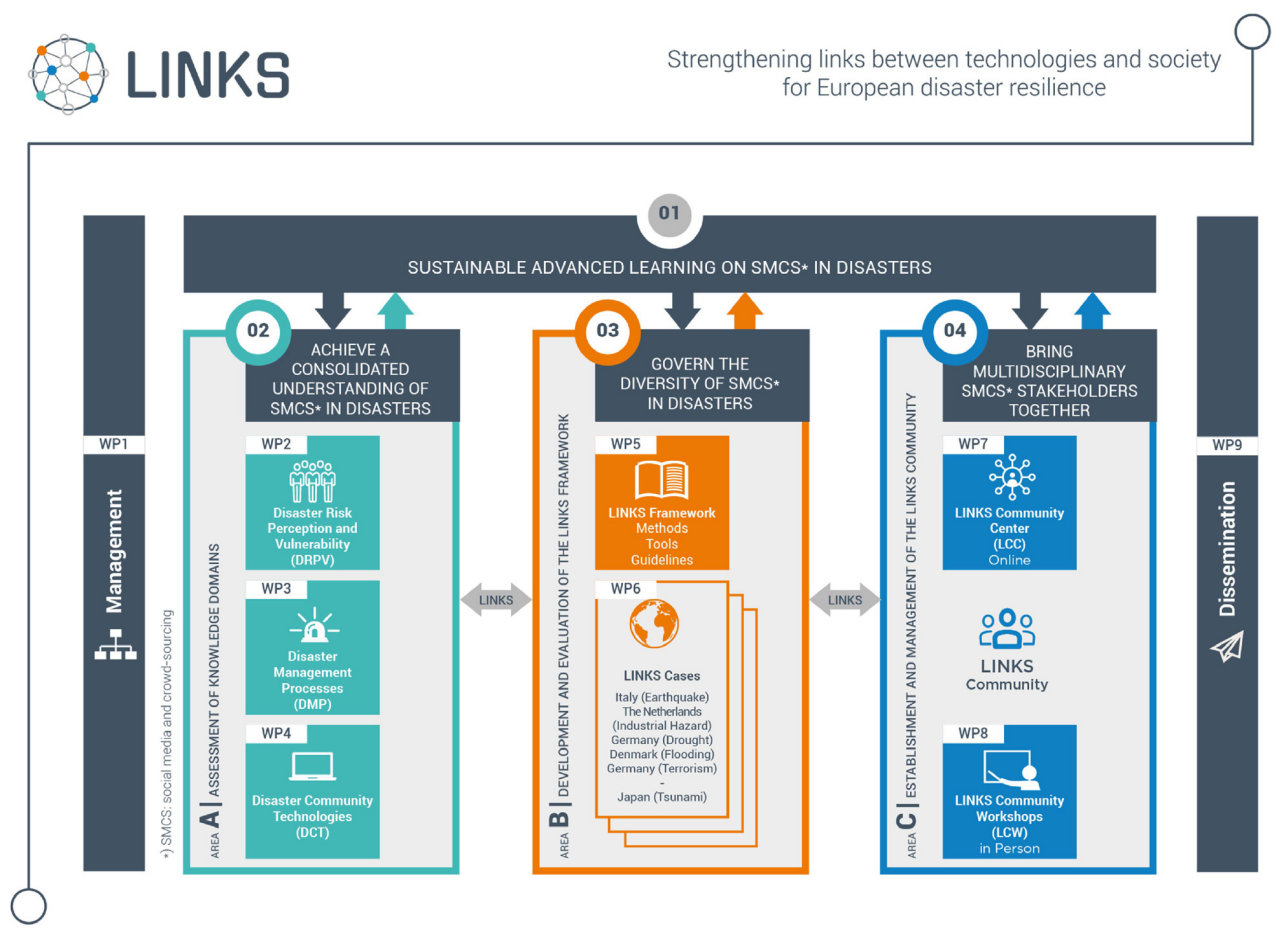
The three areas of the LINKS project.

In Figure 1, the three areas of the LINKS project, related to the objectives defined in the previous section, are represented.

The three knowledge domains are considered the essential aspects to analyze to reach the objectives of LINKS, since they represent the crucial dimensions of disaster resilience: the social, the institutional, and the technological dimensions. Investigating, through a structured review of existing literature and projects, not only the individual meaning of these dimensions but also the interactions among them, allows the adoption of a multidisciplinary approach unique to LINKS. SMCS are considered the point of conjunction between these three knowledge domains, since LINKS is investigating how the application of these tools impact on individuals and people’s perception of the risks associated to the disaster and on the conditions of social vulnerability (DRPV), on the procedures and processes of (natural, human, technical, security) disaster management (DMP), and on the functions of the technologies used by practitioners and citizens during disasters (DCT). 

The outputs of these studies have formed the LINKS knowledge bases, which represent the foundations for the project, and feed into a set of methodologies and the evaluation of the LINKS Framework, the second area of LINKS. The methodologies applied in the cases consist of grounded and participatory approaches, and encompass several methods for data collection within the research activities. These include different social scientific methods: live and digital ethnography including surveys and questionnaires, semi-structured and open-ended interviews, and computer assisted personal interviews; participatory action research (PAR) including focus groups and stakeholder engagement workshops; and the analysis of social media data. These are applied to evaluate the content and learning potentials of the Framework. 

The Framework consists of different learning materials and components, such as methods, tools, and guidelines, aimed at different stakeholders, including practitioners, researchers, policy makers, and citizens, to provide a better understanding the diversity around, and improving the application of, SMCS in disasters. This is done by sorting knowledge along themes (e.g. inclusiveness, risk communication, etc.) and learning paths within the Framework based on the objectives of different stakeholders. The foundations for the Framework are the LINKS knowledge bases, and ongoing related knowledge gathered in three iterative steps of the project. This entails case-based assessments of the Framework to validate and extend the knowledge bases across five scenarios (the LINKS cases), which differ not only in geographical characteristics and in the kind of hazards they explore, but also in socio-cultural conditions and urban dimensions. These cases cover:

- The earthquake scenario, in Italy, characterized by multi-hazards dynamics and seismic swarms, and which affect mountain areas and shrinking communities.
- The industrial hazard scenario, in The Netherlands, characterized by chemical spills which require a strong preparation of citizens.
- The drought scenario, in Germany, affecting large scale areas and characterized by water shortages and forest fires for long periods.
- The flooding scenario, in Denmark, characterized by early warnings and forecasts and which, for this reason, requires a constant flow of information and data.
- The terrorism scenario, in Germany, characterized by the lack of a good quality information and of an appropriate training to face the related issues.
- The tsunami scenario, in Japan, characterized by a low frequency and by the need of an appropriate organization to shelter people in a short time (potential case, TBD).

The third LINKS area is the LINKS Community and LINKS Community Center (LCC). In fact, according to the last objective defined in the previous section, we intend to create a multidisciplinary and sustainable community of stakeholders from several countries and professions. They will actively collaborate with the LINKS Consortium in order to learn and benefit from the project development and results, and ultimately carry on the project outcomes into the future. This participation will be enabled in two ways.

On the one hand, the LCC will be designed as an online web platform for sharing and integrating lessons learned and ongoing experiences will be created. This platform will represent a valuable tool in order to embed the LINKS Framework, obtain feedback, and engage stakeholders in a continuous dialogue. 

On the other hand, in person events will be organized. The LINKS Community workshops will aim to foster the sharing of experiences and knowledge among key stakeholders in the LINKS cases with relevant external experts and professionals. They will be held in each selected case countries and represent an essential item for evaluating the LINKS Framework. A considerable contribution to the LINKS workshop will be madeby a group of invited advisors from different relevant organizations representing practitioners, public authorities, researchers, industrial stakeholders, and citizens (the LINKS Advisory Committee), which will drive and inform LINKS during its entire life cycle.

### LINKS preliminary findings

At this stage in the project, LINKS is finalizing the reports from the assessments of the three knowledge domains and creation of the DRPV, DMP, and DCT knowledge bases. The results from the knowledge bases are being mapped to themes emerging from a number of internal workshops and meetings held with the practitioner partners in the project, to better understand their needs, experiences, and expectations in this domain, andto provide a means of operationalizing many of the concepts, assumptions, gaps and best practices identified in the individual knowledge bases. Those findings are presently being integrated into the methodologies for the first evaluation of the LINKS Framework in the cases set to begin in November 2021. Throughout the duration of the project, LINKS will continue to disseminate the project findings and outputs through various channels including the project website, seminars, conferences, DRS01 and EC related events and networks such as CERIS, the DGECHO Knowledge Network and CMINE. LINKS will also ensure that the findings are exploitable and sustainable through the design of the Framework and LCC, so that relevant stakeholders are able engage and contribute to the findings long after the project has concluded. Exploitation and sustainability plans can be found in upcoming deliverables (e.g. D7.1 and D9.2)

**Figure 2.**
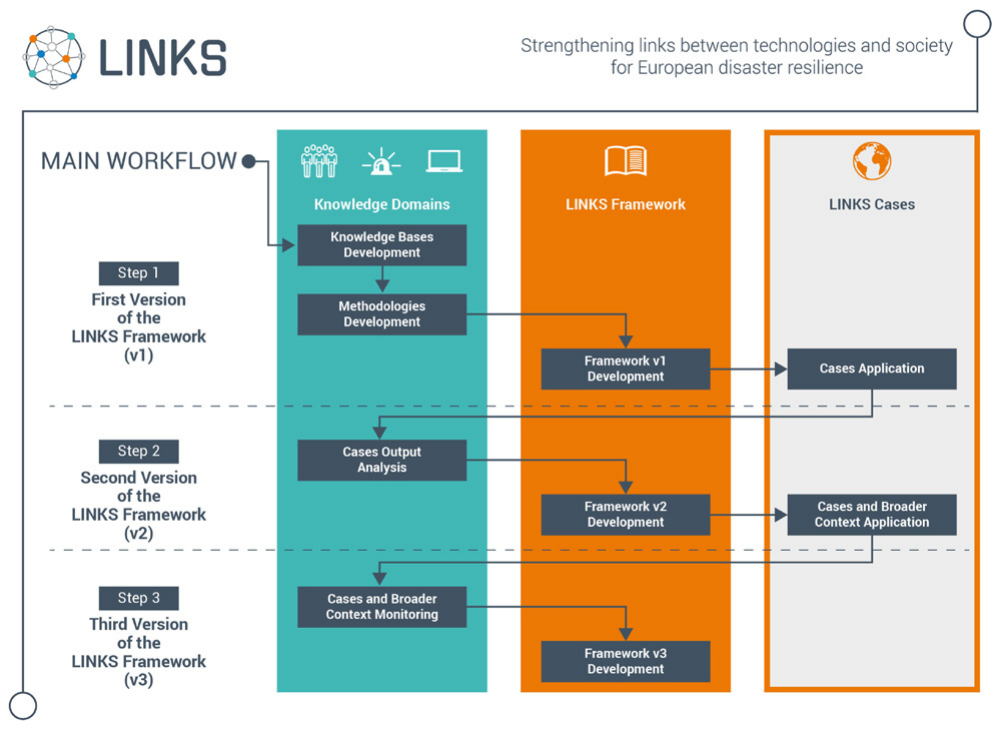
LINKS project workflow.

The current project stage is illustrated in the green box in the Step 1 row in Figure 2.

***DRPV*.** This knowledge base encompasses two key concepts related to resilience which are frequently discussed in the disaster literature. Accordingly, a literature review has been conducted on the two concepts in the context of the digital space, with the aim of understanding how social, virtual platforms (like SMCS) can interact and modify them ( Bonati, 2021; Pazzi *et al.*, 2021). Risk perception can be defined as the way people interpret reality, how they characterize and evaluate hazards, and the perceived likelihood of encountering a hazard based on their levels of knowledge ( Douglas & Wildavsky, 1982; Gierlach *et al*., 2010; Pazzi *et al*., 2016; Yong & Lemyre, 2019). On the other hand, vulnerability is a condition acquired over time and linked to the idea that a disaster can simultaneously produce experiences of vulnerability and resilience ( Fordham *et al*., 2013; Lewis, 2012; Uekusa & Matthewman, 2017). 

Results show that how we define vulnerability, and how we perceive risks, can be shaped by the way we use and interact with SMCS. This implies both potentials and limits for the transition of disaster management processes to the digital space. For instance, access to resources, information, and rescue can be facilitated through the use of social platforms in emergencies, helping to reduce vulnerabilities. However, not everyone has the same ability and possibility of accessing the information or resources, which increases the risk of individuals becoming ‘invisible’ during disasters. Furthermore, results show that the population should be educated to deal with disasters using social media. The information flow and the way in which social media are perceived by the users affect the communication from the authorities to the people and trust in the authorities. (see for example: Dressel, 2015; Jurgens & Helsloot, 2018; Kaufhold *et al*., 2019; Reuter *et al*., 2016; Wåhlberg & Sjöberg, 2000). Again, the issue of accessibility of information is relevant in the measure in which institutions are able to provide targeted communication while maintaining comprehensibility. Lastly, some limitations have been identified in the use of social media to assess risk perception and vulnerability, i.e. disinformation, fake news, and the dark net ( Von Stulpnagel & Krukar, 2018). 

In the next steps of the project, a methodology, set of tools, and guidelines will be provided to define how SMCS can increase people’s risk perception, strengthening the reciprocal trust between the policy makers, practitioners, and citizens. Moreover, we will investigate how some social groups, that usually are identified as vulnerable, can increase their capacity to deal with risks thanks to the use of digital technologies, and how they can become relevant resilient actors in the disaster management processes. 

***DMP and governance.*** Departing from the technology and disaster governance nexus, the project’s initial contributions on governance come from two research-based analyses: first, an academic literature review of social media and crowdsourcing in relation to disaster governance. Second, a mapping of existing international, European and national guidelines and policy Frameworks that currently govern the use of social media and crowdsourcing in the management of disasters. Together, these two analyses highlight that social media and crowdsourcing technologies provide immense opportunities for effective and inclusive disaster governance and management processes overall (see for example Chen & Sakamoto, 2014; Graham & Avery, 2013; Roche *et al*., 2013). Nevertheless, existing research shows that the full governance and management potential of social media and crowdsourcing platforms in disasters is underutilized ( Crowe, 2011; Graham *et al*., 2015; Harrison & Johnson, 2019). Theever increasing variety and number of stakeholders in disaster risk management ( Raju, 2013) highlights the need for capacity development within national governments, and among other actors, for the use of social media and crowdsourcing technologies in disaster risk management and the need for an even bigger call for greater integration of social media and crowdsourcing technologies in disaster risk management plans ( Carley *et al*., 2016; Graham *et al*., 2015). For these various social media and crowdsourcing platforms to play a significant role, it can be argued that this role must not only be reflected in relevant legal Frameworks, policies and guidelines but also provide clear guidance on questions of ethics. 

In the future, given the increasing presence of social media and crowdsourcing platforms in our daily lives and also during different disasters, it must be stressed that we need a more inclusive approach for use of these technological platforms. Such inclusion involves an increased focus on social media and crowdsourcing not only during various phases of the disaster management cycle but also clearly reflected in plans and policies. Furthermore, we argue the need for a deeper understanding and integration of a people-centred approach where technology culture, risk perceptions and norms are considered important for how social media and crowdsourcing can play a role in disaster governance ( Nielsen & Raju, 2021).

***DCT*.** Accompanying the other two knowledge domains, the third domain focuses on the technical perspective of SMCS. The overall objective within the knowledge domain of DCTs is to provide a consolidated understanding and overview of SMCS technologies in disaster situations. This forms the knowledge base on DCT in the project. For this purpose, a literature search was conducted. This included good practices for DCTs, the analysis of existing guidelines (e.g. Helsloot *et al.*, 2015), impacts and challenges (e.g. Gizikis *et al*., 2017) of SMCS as well as relevant IT-classifications (e.g. Schäfer *et al*., 2017). Following this, a global business market analysis of existing DCTs was carried out, resulting in a list of existing and usable DCTs and the extraction of functional and technical properties. 

The analysis leads to a basic understanding of the technological perspective on SMCS in the context of disasters and is the basis for creating the first draft of a category system, the so-called DCT-schema. The DCT-schema enables the classification and comparison of DCTs using an extensive set of categories. Features such as the functional scope of the DCTs (e.g. real-time analysis and automatic event detection) as well as technical requirements (e.g. interfaces for integration into third-party applications or the handling of metadata) are taken into account ( Habig *et al*., 2021). 

Future work within this knowledge base includes the further development of the DCT-schema within the context of the LINKS Framework, to improve the comparability of relevant DCTs. This will be done in collaboration with relevant stakeholders within the cases as well as the DRPV and DMP knowledge bases. The DCT-schema will help in the development of a methodology for the continuous assessment of SMCS technologies in different processes. This is achieved through continuous monitoring of new technologies due to an ongoing business market analysis. The necessary information for the DCT-schema will be identified with the involvement of stakeholders within the project. Plans for the future foresee the aspect of crowdsourcing to work and use the schema by anyone interested via a collaborative web-based platform – the LINKS Community Center (LCC). Currently LINKS is exploring the potentials for the LCC to interface with other relevant platforms and networks already in use in the disaster and crisis management sector, such as the CMINE (Crisis Management Innovation Network Europe) platform. 

***Case-based assessments of the LINKS Framework*.** The main objective of LINKS is to foster sustainable advanced learning through an evolving set of learning processes and materials, such as methods, tools and guidelines for governing the diversity around the use and the understanding of SMCS in all phases of disasters. Those learning elements will be included in the so-called LINKS Framework which is currently being co-designed. Ultimately, the Framework will serve different types of stakeholders (from practitioners to policy-makers). To develop the Framework in a way which is at the same time scientifically robust and grounded in the needs and challenges of potential stakeholders, both the knowledge bases and the knowledge of the stakeholders who will benefit from the Framework are taken into account. Several meetings with one stakeholder group (practitioners from four European countries) have shed light on experiences and needs regarding the use of SMCS in specific contexts. The gaps identified in the literature revolving around DRPV, DMP and DCT will serve as inputs to test initial assumptions in five cases using the scenarios mentioned above. The results will feed into the Framework and will be structured around learning objectives and ad-hoc learning materials. The latter are envisaged as a bulk of knowledge that can be “acted upon” in a dynamic way rather than just “accessed” by relevant stakeholders. What needs to be learned by whom as well as how to enable dynamic learning processes, will become clearer in the course of the project through the application in local cases. The Framework will follow a three-step iterative process and will be evaluated and refined in two rounds of case-based assessments. The final version will be ready for the wider crisis management community in 2023. 

## Conclusions

This paper has provided an overview of the research presently being conducted by the LINKS Horizon 2020 project. The work in the project stems from an understanding of the challenges faced by communities attempting to utilize SMCS technologies and solutions in an effort to prevent, mitigate, respond to and recover from the damaging effects of hazards, be they of natural or human-made origins. These processes provide great potential for sharing critical information and for capitalizing on the knowledge and experiences of different actors in times of crises. Nevertheless, the diversity surrounding the implementation and use of SMCS also creates uncertainty among institutions and individuals as to the efficacy and best practices for these solutions. Moreover, data and technology overload, false information and misinformation, ethics and privacy issues, and the lack of accessibility by some of the most vulnerable groups create additional barriers in this area. 

The LINKS project aims to govern this diversity by creating a living repository of diverse knowledge on SMCS in disasters. The project has already identified key gaps, needs, best practices and themes cross the knowledge domains of DRPV, DMP and DCT and is set to explore and test the assumptions derived from these domains and the experiences from our practitioner partners in a series of upcoming case-based assessments across Europe. The preliminary findings across the knowledge domains have exposed the ways in which fluid dimensions of diversity, accessibility, connectivity, and mobility, as well as individual and environmental factors, may influence the ways in which vulnerabilities and risk perceptions effect resilience. The findings have further identified common themes across the knowledge domains and workshops with practitioners, relating to trust and managing misinformation, gaps in the inclusiveness of vulnerable groups and engagement with citizens, and the need to better understand the technical and practical approaches for effective risk communication among communities. These findings have established the project’s knowledge bases, and together with case findings will be the foundation for developing an interactive Framework which enables learning of the SMCS/disaster issues for different stakeholders. Developments around SMCS and disasters change and evolve as quickly as the underlying technology itself. It is therefore important that the Framework enables learning which can keep up and adapt with the changes (advanced) and be sustainable. In this regard, and through this learning, LINKS sees the potential to grow a specific community of stakeholders that can learn from and contribute their diverse knowledge and experience within the community. Ultimately, this project seeks to enable communities to harness the full potential of SMCS in all phases of disasters, and thereby strengthen their resilience. 

## Data availability

No data are associated with this article.
