# Supplementary material for: Exploring the impacts of social media and crowdsourcing on disaster resilience
Source: Open Res Eur. 2024 Jan 5;1:60. Originally published 2021 Jun 1. [Version 3] doi: 10.12688/openreseurope.13721.3 (PMC10877096; doi:10.12688/openreseurope.13721.3)
Supplement: Peer review history

# Reviewer report: Reviewer response for version 3

**DOI:** 10.21956/openreseurope.17976.r37281

**Published:** 2024-02-19

**Authors:** Pakhee Kumar

**Affiliations:** 1 Institute for Sustainable Heritage, University College London, London, UK

Many thanks to the authors for editing the paper and their response.

Is the study design appropriate for the research question?

Yes

Are sufficient details of the methods provided to allow replication by others?

Partly

Is the rationale for, and objectives of, the study clearly described?

Yes

Are the datasets clearly presented in a useable and accessible format?

No

Reviewer Expertise:

Disasters, social media, crowdsourcing, cultural heritage

I confirm that I have read this submission and believe that I have an appropriate level of expertise to confirm that it is of an acceptable scientific standard.

# Reviewer report: Reviewer response for version 2

**DOI:** 10.21956/openreseurope.17029.r30991

**Published:** 2023-06-22

**Authors:** Pakhee Kumar

**Affiliations:** 1 Institute for Sustainable Heritage, University College London, London, UK

As mentioned in my previous report, the authors have written an interesting article about a very timely project. However, the comments I provided in version 1 don't seem to be addressed in version 2. Even the typos from version 1 are present in version 2.

 Therefore, I request the authors to provide a response to the points.

Is the study design appropriate for the research question?

Yes

Are sufficient details of the methods provided to allow replication by others?

Partly

Is the rationale for, and objectives of, the study clearly described?

Yes

Are the datasets clearly presented in a useable and accessible format?

No

Reviewer Expertise:

Disasters, social media, crowdsourcing, cultural heritage

I confirm that I have read this submission and believe that I have an appropriate level of expertise to confirm that it is of an acceptable scientific standard, however I have significant reservations, as outlined above.

# Reviewer report: Reviewer response for version 2

**DOI:** 10.21956/openreseurope.17029.r30992

**Published:** 2023-03-13

**Authors:** Rob Grace

**Affiliations:** 1 Technical Communication & Rhetoric, Texas Tech University, Lubbock, TX, USA

No additional comments.

Is the study design appropriate for the research question?

Yes

Are sufficient details of the methods provided to allow replication by others?

Partly

Is the rationale for, and objectives of, the study clearly described?

Partly

Are the datasets clearly presented in a useable and accessible format?

Yes

Reviewer Expertise:

Information science, crisis informatics, risk communication

I confirm that I have read this submission and believe that I have an appropriate level of expertise to confirm that it is of an acceptable scientific standard.

# Reviewer report: Reviewer response for version 1

**DOI:** 10.21956/openreseurope.14796.r28035

**Published:** 2021-11-30

**Authors:** Rob Grace

**Affiliations:** 1 Technical Communication & Rhetoric, Texas Tech University, Lubbock, TX, USA

**Summary**

 The paper introduces the LINKS Project, which seeks to improve European disaster resilience by communicating best practices for the use of social media and crowdsourcing (SMCS) in disaster management. To do this, the project will 1) assess existing knowledge related to disaster risk perception and vulnerability, disaster management processes, and disaster community technologies; 2) develop and evaluate a framework that organizes knowledge on tools, methods, and guidelines related to the use of SMCS for disaster management; and 3) share these findings with relevant stakeholders through an online platform and in-person events. To inform the establishment of best practices, the project will examine six case studies of SMCS in disaster management in multiple European countries and Japan.

**Review**

 The authors take up the very difficult challenge of summarizing diverse literatures, examining multiple case studies, and specifying sets of best practices relevant to public, government, and industry stakeholders. These efforts stand to make important contributions to our understanding of how we should employ crowdsourcing and social media tools and practices during disasters. 

 However, while the project’s breadth is its primary strength, this breadth also makes it difficult to clearly summarize and communicate insights drawn from diverse knowledge bases and case studies. The following comments discuss the need for a clear, high-level framing of the findings presented in the paper.

 The paper begins by defining crowdsourcing, the focus of the project, as the act whereby an entity (i.e., crowdsourcer) outsources a task, typically via an open call, to a crowd of volunteers (i.e., crowdworkers). Does this definition correspond with the general focus of the project? If so, it would seem to offer an opportunity to organize the paper according to the crowdsourcers, tasks, types of calls, and crowdworkers that characterize uses of SMCS for disaster management. 

 The high-level framework would help clarify the actual uses of SMCS that the project is investigating. While the project is ongoing and the presentation of findings is preliminary, readers are likely to find these uses vague when reading the current draft. Diverse and concrete uses of SMCS could be outlined for the many general use contexts presented in the paper: disaster awareness, early warning, disaster risk reduction, rapid damage assessment, etc. This will no doubt be important when developing a framework that will organize the best practices and “common themes” discussed in the conclusion. This framework will likely need to address, in some form, who is using this information (e.g., crowdsourcers), how they are gathering the data (e.g., type of call), who is generating the data (e.g., crowdworkers), and the disaster management activities that are facilitated by these kinds of work arrangements (e.g., tasks).

 Similarly, it remains unclear how the case studies will inform the framework of best practices with respect to the three areas of concentration: disaster risk perception and vulnerability, disaster management processes, and disaster community technologies. Will each case focus on one of these concentrations or address aspects of all three?

 Understanding the provisional nature of the draft and its findings, these comments are offered to recommend approaches the authors can take to clearly communicate the breadth of findings related that can explain, with specificity, how SMCS tools/practices have been and should be used for disaster management.

 Overall, the authors deserve congratulations for advancing this challenging and important research.

**Minor**:

 The paper variously refers to SMCS and SCMS.

 In the introduction, the clause “be it based on local activities, or globally connected” requires revision.

 The following sentence requires revision: “They furthermore recognize the reward of undertaking the tasks for the crowd, e.g. citizens, as it contributes to knowledge and experience, self-esteem and resources, and the benefit for the crowd sourcer in terms of the utilization of what the user brought to the table.” Perhaps, “Crowdsourcers benefit by accomplishing outsourced tasks while crowdworkers receive rewards in the form of  knowledge, experience, increased self-esteem, and material resources."

 The line “achieving a consolidating understanding of SCMS in disasters” should be rewritten as “achieving a consolidated understanding of SMCS in disasters.”

Is the study design appropriate for the research question?

Yes

Are sufficient details of the methods provided to allow replication by others?

Partly

Is the rationale for, and objectives of, the study clearly described?

Partly

Are the datasets clearly presented in a useable and accessible format?

Yes

Reviewer Expertise:

Information science, crisis informatics, risk communication

I confirm that I have read this submission and believe that I have an appropriate level of expertise to confirm that it is of an acceptable scientific standard, however I have significant reservations, as outlined above.

# Reviewer report: Reviewer response for version 1

**DOI:** 10.21956/openreseurope.14796.r27008

**Published:** 2021-07-14

**Authors:** Pakhee Kumar

**Affiliations:** 1 Institute for Sustainable Heritage, University College London, London, UK

The authors have written an interesting paper on the LINKS project funded by the European Commission under the Horizon 2020 Research and Innovation Programme. The contribution to knowledge is very clear and has implications for a wide section of society. I enjoyed reading this paper and appreciate the effort that has gone into it, particularly in developing the process illustrated in Figures 1 and 2. There are a few areas that need strengthening:   

1. This paper has created a new terminology SMCS combining two concepts: social media and crowdsourcing. This needs to be delineated as social media and crowdsourcing are different concepts with some overlaps. The authors should explain in brief the theoretical underpinning of SMCS. This reference could be a good starting point for social media:  Kaplan, A.M. and Haenlein, M., 2010. Users of the world, unite! The challenges and opportunities of Social Media. *Business horizons*, *53*(1), pp.59-68.
2. In the Introduction, the authors have highlighted the use of crowdsourcing platforms during disasters with examples such as Ushahidi. However, it is not clear which social media platforms are used extensively during disasters and how.
3. It would also be useful to highlight that the research will be focusing on participatory technologies that are facilitated by the internet and Web2.0. Participatory methods (including crowdsourcing in disaster context) were used before the internet age also, so I believe that it is worth clarifying.
4. The term DCT is introduced early on, however, it is explained quite late in the paper. It makes understanding the concept a little difficult, particularly because it is not a widely used term. Moreover, I would suggest that the authors reconsider the use of the term “Disaster Community Technologies”. On page 7, the authors have mentioned that in the DCT section the aim is to provide an overview of SMCS technologies. In my view, coining a new term for this is quite unnecessary. As mentioned before, the terminologies should be delineated if used.
5. I suggest that the authors consider including a list of LINKS partners in the appendix. It would be useful for the readers to see the distribution across the categories. If not names, consider including their roles as per the five categories defined on page 4.
6. In the LINKS approach (mentioned on page 4), will the authors also include the analysis of crowdsourced data? In para 3, only analysis of social media data is mentioned.
7. The LINKS cases are interesting and relevant to develop the framework. However, it is not clear how these scenarios were developed. Was it based on the frequency of events in the geographical region or the risk of such events? Moreover, the geographical spread is a bit uneven with most of the cases in Europe and a potential case in Japan. Is it possible to diversify the geographical locations? Is it possible to include more scenarios (e.g. fire)?
8. LCC online platform will indeed be useful for the stakeholders. One thing to consider is: how will it be updated and upgraded once the project ends? This is necessary due to the fast-changing pace of SMCS.
9. DCT section on page 7 needs more clarity. For instance, automatic classification is not an integral part of SMCS but developed by researchers due to 1) use of social media by citizens 2) the architecture of social media which affords instantaneous news sharing. Also, the role of crowdsourcing is not clear in this section.
10. There are some typos in the document. E.g. SCMS instead of SMCS on page 4. I also suggest rephrasing some sentences. E.g. ‘thanks to the use of digital technologies’ on page 7.

Is the study design appropriate for the research question?

Yes

Are sufficient details of the methods provided to allow replication by others?

Partly

Is the rationale for, and objectives of, the study clearly described?

Yes

Are the datasets clearly presented in a useable and accessible format?

No

Reviewer Expertise:

disasters, social media, crowdsourcing, cultural heritage

I confirm that I have read this submission and believe that I have an appropriate level of expertise to confirm that it is of an acceptable scientific standard, however I have significant reservations, as outlined above.

## References

[1] : Users of the world, unite! The challenges and opportunities of Social Media. *Business Horizons* .2010;53(1) : 10.1016/j.bushor.2009.09.003 59-68 10.1016/j.bushor.2009.09.003
